# Future perspectives on the use of molecular assays for pathogen identification in neonatal sepsis: a survey study among members of the European Society for Paediatric Infectious Diseases

**DOI:** 10.1136/bmjpo-2026-004600

**Published:** 2026-04-30

**Authors:** Jip Groen, Martijn van der Kuip, Marc A Benninga, Tim de Meij, Hendrik J Niemarkt

**Affiliations:** 1 Paediatric Gastroenterology, Amsterdam UMC, Amsterdam, Netherlands; 2 Amsterdam Reproduction and Development Research Institute, Amsterdam, The Netherlands, Amsterdam, Netherlands; 3 Paediatric Infectious Diseases, Rheumatology and Immunology, Amsterdam UMC, Amsterdam, Netherlands; 4 Neonatology, Máxima Medisch Centrum, Veldhoven, Netherlands

**Keywords:** Neonatology, Microbiology, Intensive Care Units, Neonatal

 Neonatal sepsis (NS) is a major cause of morbidity and mortality yet challenging to diagnose due to non-specific symptoms and blood culture (BC) turnaround time.^1^,^3^ Diagnostic uncertainty prompts high rates of empiric antibiotic treatment, with many neonates exposed to prolonged therapy despite sterile BCs, specifically for early-onset NS.^4 5^ Molecular assays have been proposed to accelerate pathogen identification and support antibiotic stewardship.^6^ However, large-scale implementation has yet to follow. Understanding clinician perspectives is essential to guide future diagnostic implementation.

We conducted a cross-sectional, anonymous, web-based survey (EUsurvey, European Commission) (blank version, online supplemental file 1) disseminated via the European Society for Paediatric Infectious Diseases (ESPID) between April 2024 and March 2025. The survey was piloted by 10 physicians with relevant expertise for content and time consumption (mean 13 min to complete, range 10–18 min). The ESPID membership base (n=2701) was addressed through three emails in April 2024, September 2024 and January 2025. Survey invitations explicitly mentioned NS expertise as entry criterion but this could not be guaranteed. The anonymous nature of the survey restricted prevention of double entries. The survey focused on experiences with BC and expectations regarding molecular diagnostics, implementation barriers and case scenarios. Quantitative data were analysed descriptively, and free-text responses were analysed thematically by two independent coders. Reflexive thematic analysis was applied and consensus was reached through discussion. All analyses represent crude values. Fisher-Freeman-Halton exact tests were used to compare proportions.

Of 187 respondents accessing the survey, 180 completed >70% of items in the outcome domain (response rate 7%).

43% anticipated that molecular assays will fully replace BCs as the primary pathogen ID test for NS within the next 20 years. Medical microbiologists were least likely to anticipate full replacement (27%), although subgroup sizes were small. Among respondents anticipating a complementary role, the reported barriers to implementation are displayed in table 1. Notably, medical microbiologists frequently raised concerns about antimicrobial resistance (AMR) profiling but did not express concerns regarding diagnostic accuracy.

**Table 1:** Survey-reported concerns regarding the use of molecular assays in neonatal care

Thematic arguments	(n=100) (%)
Lack of adequate antimicrobial resistance profiling in molecular assays	44 (44%)
Suboptimal diagnostic accuracy of molecular assays	31 (31%)
Uncertainty regarding broad range application of molecular assays across clinical indications	14 (14%)
Cost efficiency issues with molecular assays	10 (10%)
Transition to routine use of molecular assays as primary modality needs more than 20 years	5 (3%)
Transition to molecular assays will require skill and resources not available in some settings	6 (6%)
Molecular assays provide limited potential to subtype pathogens following genetic mutations and during outbreak management	3 (3%)
Lack of species identifying capacity of molecular assays	2 (2%)
Lack of automation of diagnostic workflow for molecular assays*	1 (1%)
Environmental impact of molecular assays	1 (1%)

* Lack of automation was explained by referencing automated blood culture systems that require very little handling, especially in culture negative cases

Furthermore, 49% of respondents reported experience with BC-negative NS requiring antibiotic treatment prolongation, and 36% were unsure. Participants guessed that on average 28 out of 100 neonates treated for suspected sepsis may suffer BC-negative NS, which is roughly in agreement with a previous study.^4^ Neonatologists had significantly lower estimates than other professionals (18 vs 31 per 100; p<0.01). Professionals in high-resource settings estimated fewer BC-negative cases than those in mixed or low-resource settings (24 vs 36 vs 46 per 100, respectively; p<0.001). It should be noted that culture-negative cases can reflect either truly missed cases, suboptimal blood collection conditions, non-bacterial aetiologies or the result of unnecessary treatment prolongation.

Participants on average indicated that a turnaround time of 13 hours would be acceptable and desirable for a novel diagnostic assay, assuming diagnostic performance equivalent to BC. With access to a hypothetical molecular assay offering <4-hour results and 100% sensitivity compared with BC, respondents were willing to tolerate a mean specificity loss of 19%. 18% of participants did not answer this question. Missing responses were not related to any of the baseline variables shown in table 2 and we suspect the complexity of the question led some participants to refrain from answering. As this variable reflects subjective estimates obtained through expert elicitation rather than unobserved true values, missing responses were not imputed. The most frequently reported motivations for accepting lower specificity were quicker diagnosis to improve prognosis (73%) and shortening of hospital admission and antibiotic courses (54%). In a hypothetical scenario involving a clinically well-appearing neonate with suspected early-onset NS and access to an assay offering <4 hour results with identical test performance to BC, 73% of respondents indicated they would postpone antibiotic treatment until the results were available. In a second scenario assessing treatment (de)escalation after prior antibiotic initiation, 61% would stop antibiotics if Molecular assay results were negative and the neonate appeared well, whereas 39% would continue treatment, most commonly until inflammatory markers were available or 24–48 hours of treatment had passed as a safety margin. No significant differences were found for all reported outcomes in early-versus-late wave responses, in which time cohorts were categorised based on the timing of responses relative to email invitations.

**Table 2:** Participant characteristics of survey respondents

Participant characteristics	All (n=180) (%)
Professional title*	
Paediatric infectious diseases specialist	74 (41.1%)
Neonatologist	40 (22.2%)
General paediatrician	34 (18.9%)
Paediatric resident	12 (6.7%)
Medical microbiologist	11 (6.1%)
Researcher in paediatrics	6 (3.3%)
Other†	3 (1.7%)
Based in	
Europe	145 (80.6%)
Asia	14 (7.8%)
Africa	7 (3.9%)
North America	6 (3.3%)
South America	5 (2.8%)
Oceania	3 (1.7%)
Resource setting‡	
High resource setting	122 (67.8%)
Mixed resource setting	36 (20%)
Low resource setting	22 (12.2%)
Primary work environment*	
Academic hospital	108 (60%)
Non-academic hospital	58 (33.3%)
Community outpatient clinic	8 (4.4%)
Clinical working experience	
0–10 years	53 (29.4%)
11–20 years	69 (38.3%)
21–30 years	33 (18.3%)
31–40 years	23 (12.8%)
41–50 years	2 (1.1%)

* When professionals self-identified as more than one category, they were asked to select the title that best reflected the majority of their activities.

† Other professionals identified as a paediatric gastroenterologist, a paediatric emergency doctor and a clinical pharmacologist with bias in paediatric infectious diseases.

‡ No definitions for resource setting were provided to respondents.

These findings suggest that the participating clinicians perceive substantial potential for molecular diagnostics to improve NS management. However, the results should be interpreted cautiously because of a low response rate, combined with ESPID-based sampling that predominantly reflects European and high resource settings, introducing a substantial risk of selection bias and limiting the external validity.

Crucial barriers to adoption of molecular assays remain, especially regarding AMR profiling and diagnostic accuracy. While shorter treatment durations seem achievable with proper implementation, a considerable proportion of survey participants prefer to retain the traditional treatment window of 24–48 hours as a safety margin that is inherent to BC turnaround, highlighting that technological advances alone may not solve the full equation. Future development of diagnostics should prioritise same-shift turnaround, integrated AMR and robust validation studies to support safe implementation. Additionally, antibiotic stewardship, culture change and risk tolerance should be considered. Addressing these challenges will ensure that molecular diagnostics meaningfully contribute to improved NS care.

## Supplementary material

10.1136/bmjpo-2026-004600 online supplemental file 1
