# Supplementary material for: Future perspectives on the use of molecular assays for pathogen identification in neonatal sepsis: a survey study among members of the European Society for Paediatric Infectious Diseases
Source: BMJ Paediatr Open. 2026 Apr 30;10(1):e004600. doi: 10.1136/bmjpo-2026-004600 (PMC13141089; doi:10.1136/bmjpo-2026-004600)
Supplement: online supplemental file 1 [file bmjpo-10-1-s001.pdf]

Drivers and barriers of implementation of novel diagnostic strategies in neonatal sepsis management

Fields marked with * are mandatory.

Introduction - Survey on the use of novel diagnostic modalities for pathogen identification in neonatal sepsis management

This questionnaire is **fully anonymous.** We will not ask any questions that allow personal identification and EUSurvey does **not store your IP address**. **WE INFER THAT YOU CONSENT to using the generated data once you have completed the survey.** After completion it is not possible to retract your entry.

This survey is not aimed at reaching any type of consensus, rather it intends to inform the scientific community on the attitudes of pediatric health care professionals and microbiologists towards (future) use of novel diagnostic techniques for pathogen identification in neonatal sepsis. For the questions asked in this survey there is no right-or-wrong.

The primary aim of the survey is to allow a comprehensive display of desired qualities of novel diagnostic techniques, as well as to identify potential barriers to implementation of such techniques. This information may support health care policy makers during implementation of novel diagnostic strategies in the future. To enhance our understanding of the different perspectives on this topic, discriminatory questions on your professional activities (e.g. resource setting and subspecialty training) and experience are asked to display the survey results from various relevant perspectives.

This survey will take up approximately 10-15 minutes of your time.

For any questions regarding the survey content you may contact the coordinator through j.
groen5@amsterdamumc.nl

Demographic data

What title fits best with your professional activities

- ☐ General Pediatrician
- ☐ Neonatologist
- ☐ Pediatrician - Infectious diseases specialist
- ☐ Medical microbiologist
- ☐ Pediatric resident (either training or non-training)
- ☐ Researcher in pediatrics
- ☐ Any other, please specify below

Specify your profession if not included in the list above

75 character(s) maximum

In what part of the world do you predominantly practice medicine?

- ( ) Europe
- ( ) Asia
- ( ) Africa
- ( ) North America
- ( ) South America
- ( ) Oceania

In your opinion, in what kind of setting do you practice medicine predominantly?

- ( ) Low resource setting
- ( ) Mixed resource setting
- ( ) High resource setting

How would you describe your current clinical environment?

- [ ] Nonacademic hospital
- [ ] Academic hospital
- [ ] Community outpatient clinic
- [ ] Indenpendent diagnostics center

How many years of clinical experience do you have?

- ( ) 0-10 years
- ( ) 11-20 years
- ( ) 21-30 years
- ( ) 31-40 years
- ( ) 41-50 years

How often are you involved in the management of neonatal sepsis?

Maximum 1 selection(s)

- [ ] Never
- [ ] Rarely
- [ ] Sometimes
- [ ] Often
- [ ] Weekly

Novel pathogen identification techniques

Context

In current practice, health care professionals may choose from several diagnostic modalities to support diagnosis of neonatal sepsis, such as microbiological culture, inflammatory parameters and PCR amongst many others. Most health care institutions predominantly rely on culture based approaches for patohgen identification. The disadvantage of culture based techniques is a long turnaround time. The decision to use

a culture based assay often implies simultaneous initiation of empiric antibiotic treatment, which is often continued at least up to the point of confirmed negative culture results.

Several novel molecular techniques have been developed for pathogen identification, including broad range PCR based approaches and next generation sequencing solutions, amongst many others. These diagnostic techniques offer potential for shortening the diagnostic delay of culture based assays and may have enhanced diagnostic accuracy. However, these techniques have limitations of their own.

Q: In your opinion, what will be the role of novel diagnostic techniques for pathogen identification for the diagnosis of neonatal sepsis in the coming 20 years of health care practice?

- Full replacement of the blood culture
- Addition to the blood culture, please specify below

* Please specify why you do not expect replacement of culture based assays and how these novel techniques may otherwise complement culture assays in the coming 20 years...

Time to result improvement

Context

In most health care institutions, time to result of a culture based assay varies (excluding susceptibility profiling) between 24-72 hours.

Q: If an alternative diagnostic technique is developed for pathogen identification in neonatal sepsis diagnosis, with identical diagnostic performance (i.e. sensitivity, specificity), how fast following sample retrieval should this new technique deliver results, before you would consider implementation in your hospital? Please only consider pathogen identification functionality and disregard the antibiotic sensitivity profiling for this question.

Q: Do you believe that a short course of antibiotics (i.e. 36-72 hours) prior to culture assay results may sometimes effectively treat culture negative neonatal sepsis / bacteremia cases? In other words, do you believe that some of the suspected cases that receive short antibiotic courses would have clinically deteriorated, if it were not for the short treatment they received?

- Yes
- No
- Maybe

Q: As an instictive guess, for every 100 culture negative neonates who are started on antibiotics for suspected sepsis, how many sepsis/ bacteremia cases remain 'under the radar' of detection by culture (i.e. false negative)?

Sensitivity / Specificity

Context
Most novel diagnostic assays aim for a faster turnaround time than microbiological culture. Therefore, their implementation may support decreased duration of empiric antibiotic courses following faster de-escalation of therapy after negative results. A majority of novel diagnostic techniques operates on finding genetic material of pathogens, thereby not differentiating between active and inactive or dead material. This may increase false positives that lead to a paradoxical increase in empiric antibiotic treatment courses.

Q: Assume implementation of a novel diagnostic technique for pathogen identification. Given a decrease in time-to-result from 24-72 to 4 hours (i.e. from blood culture to the unspecified novel diagnostic technique) and a hypothesized sensitivity of 100% (i.e. no false negatives) of the novel technique for neonatal sepsis, how much specificity loss (i.e. increase of false positives) would you be willing to accept as a price for a faster diagnosis and no missed cases?

Example: assume that a group of 100 neonates are tested for sepsis using a culture assay, none of them is diagnosed with true culture confirmed sepsis ultimately. 80% has a true negative culture and 20% a false positive culture (i.e. 80% specificity). Assume that the same group is tested with a novel technique. 60% has a true negative result and 40% a false positive result (i.e. 60% specificity). This is a 20% decrease in specificity. You would find additional false positive test results for 20 neonates in every 100 tested suspected cases, when using the novel technique as compared to a culture assay.

Only values between 0 and 100 are allowed

[ ] % specificity LOSS

Q: What would be your primary motivation to accept any decrease of specificity for a given novel diagnostic technique in the face of increased sensitivity and shorter diagnostic times?

(you may choose multiple answers)

- [ ] Decrease in disruption of resident and forming microbiota (intestinal, skin etc.) and sequelae in children
- [ ] Decrease in number of missed pathogens in true sepsis / bacteremia cases
- [ ] Shorter clinical admission duration (for patients without other issues and potentially for parents)
- [ ] Faster identification of pathogenic microbial species for faster treatment targeting, allowing better a prognosis
- [ ] Any other reason, please specify below

* Please specify your motivation

Case scenario 1

Assume that a novel diagnostic technique for pathogen identification in blood is implemented in your hospital today. Assume that test characteristics (sensitivity, specificity) are exactly similar to those of culture based techniques. However, this novel technique is much faster and can present a test result in 4 hours, instead of 24-72 hours.

A term newborn infant is admitted after birth to your clinic after risk assessment for early onset sepsis. During labor, mother had developed a fever up to 38.4 degrees celsius without clinical signs of infection and without an epidural in place. Membranes had been ruptured for 27 hours at the time of delivery. Maternal GBS status is unknown, no antibiotic prophylaxis was given during birth. Physical examination shows a well appearing infant directly postpartum. A blood sample is collected and the novel diagnostic technique is performed. Assume the infant remains clinically stable.

Q: What is your next step?

- ○ I initiate empirical antibiotic treatment pending the results of the diagnostic technique in 4 hours.
- ○ I order continuous monitoring of vitals but postpone initiation of antibiotic treatment until results of the diagnostic technique are available in 4 hours.
- ○ Other, please specify below

Describe your actions

Case Scenario 2

Assume that a novel diagnostic technique for pathogen identification in blood is implemented in your hospital today. Assume that test characteristics (sensitivity, specificity) are exactly similar to those of culture based techniques. However, this novel technique is much faster and can present a test result in 4 hours, instead of the usual 24-72 hours.

A term newborn infant is admitted after birth to your clinic after risk assessment for early onset sepsis. During labor, mother had developed a fever up to 38.4 degrees celsius without clinical signs of infection and without an epidural in place. Membranes had been ruptured for 27 hours at the time of delivery. Maternal GBS status is unknown, no antibiotic prophylaxis was given during birth. Physical examination of the newborn infant shows no major issues except for respiratory distress with tachypnea of 75/minute, which was present from delivery onwards. A blood sample is collected and empirical antibiotics are initiated. 4 hours later, the novel diagnostic technique delivers a negative result. Respiratory distress has disappeared completely. One dose of antibiotics has been given at that time. No inflammatory marker assay results are available at that time.

Q: What is your next step?

- ○ I discontinue antibiotic treatment and discharge the patient home (given no other issues and mother is well enough)
- ○ I discontinue antibiotic treatment and keep the patient for clinical monitoring until 24 hours postpartum have passed before I discharge
- ○ I continue antibiotic treatment until the first results of inflammatory marker assays are available and affirm lack of inflammation
- ○

I continue antibiotic treatment until at least 36 hours of treatment have passed, because with this risk profile and subtle symptoms, I do not feel fully comfortable that this infant is free of illness. The initial hours of treatment may have masked further clinical deterioration.

- Other, please specify below

Describe your actions

Comments or suggestions

Are there any comments or suggestions you wish to express regarding the contents of this survey or otherwise?
