# Supplementary material for: Future perspectives on the use of molecular assays for pathogen identification in neonatal sepsis: a survey study among members of the European Society for Paediatric Infectious Diseases
Source: BMJ Paediatr Open. 2026 Apr 30;10(1):e004600. doi: 10.1136/bmjpo-2026-004600 (PMC13141089; doi:10.1136/bmjpo-2026-004600)
Supplement: Supplementary file 2 [file bmjpo-2026-004600.reviewer_comments.pdf]

PEER REVIEW HISTORY

BMJ Paediatrics Open publishes all reviews undertaken for accepted manuscripts. Reviewers are asked to complete a checklist review form and are provided with free text boxes to elaborate on their assessment. These free text comments are reproduced below.

ARTICLE DETAILS

**TITLE (PROVISIONAL)**	Future perspectives on the use of molecular assays for pathogen identification in neonatal sepsis: a survey study amongst members of the European Society for Paediatric Infectious Diseases
**AUTHORS**	Groen, Jip<br>van der Kuip, Martijn<br>Benninga, Marc A.<br>de Meij, Tim<br>Niemarkt, Hendrik J.

VERSION 1 – REVIEW

**REVIEWER NAME**	Cabral, Bernardo
**REVIEWER AFFILIATION**	
**REVIEWER CONFLICT OF INTEREST**	No Conflict of Interest
**AI DISCLOSURE**	No, I have not used AI
**DATE REVIEW RETURNED**	20-Feb-2026

**GENERAL COMMENTS**	The survey is well designed, and the findings are of interest. Although space is limited in a research letter, the survey process should be reported in greater detail to improve reproducibility and interpretability. Please describe the sampling frame and recruitment in more detail (which ESPID channels were used, how many individuals were reached and considered eligible, whether reminders were sent, and what measures were taken to prevent duplicate responses), and report the number of complete versus partial responses together with item-level missingness. Given the 7% response rate, include a brief, explicit discussion of nonresponse/selection bias, how the respondent profile (region, setting, specialty) may differ from the target population, and simple diagnostics such as comparing early versus late responders (if feasible). Also, provide clearer information on survey administration (platform, approximate completion time, whether questions were mandatory, any randomization or branching logic, and how a single response per participant was ensured).

**REVIEWER NAME**	Kopsidas, Ioannis
**REVIEWER AFFILIATION**	
**REVIEWER CONFLICT OF INTEREST**	No Conflict of Interest
**AI DISCLOSURE**	Language editing on the review I prepared.
**DATE REVIEW RETURNED**	08-Mar-2026

**GENERAL COMMENTS**	Thank you for allowing me to review the research letter “Future perspectives on the use of molecular assays for pathogen

identification in neonatal sepsis: a survey study amongst members of the European Society for Paediatric Infectious Diseases” by Groen et al.

The manuscript addresses a gap in neonatal sepsis diagnostics.

Study design

P2 L31 April 2024-March 2025 is a very extended period for a survey. Was this sent once in April 2024 and were expecting answers until March 2025? Was it sent more than once? If that is the case, was there a mechanism that ensured that the same person did not answer the survey twice?

It is mentioned that professionals “involved in neonatal sepsis management were invited”. I would assume that ESPID does not have a way of identifying among its members who are involved in management of neonatal sepsis, and the wording of the survey does not deter someone not involved in neonatal sepsis management from answering it. This I believe needs to be under limitations.

Were there any pilot runs of the Survey?

P2 L34 L33 – “quantitative data were analysed descriptively and free-text responses thematically”

a) You do report p values. Briefly describe statistical tests used. Were there any adjustments made?

b) Thematic analysis: How were themes generated, how many coders were involved? Was inter-rater agreement assessed? Provide some information even in one sentence.

P2 – L35 Consider adding a short statement on how you managed missing data, how many responses were partially vs fully completed and any exclusion criteria

P2 L36 – 7% : even though it can be calculated please add number of contacted members.

Limitations : Please discuss in more depth the implications of the low response rate and ESPID-based sampling for selection bias and external validity, especially given the predominance of European, high-resource, academic respondents.

Findings overall should be presented with the perspective that can be given, considering the methodology and wording of the survey.

Example: P2L14 “These findings suggest that clinicians recognize the potential of molecular diagnostics to improve neonatal sepsis management”

This should reflect that these are self-reported expectations from a selected sample rather than evidence that molecular diagnostics actually improve outcomes.

Example: P3 L17 It is reported that 43% anticipate full replacement of blood culture within 20 years. However, it should be mentioned that current molecular assays generally do not provide full AMR profiling, genetic markers of resistance are not true phenotypic resistance and and may have limited pathogen range. So, such replacement expects that there will be substantial technological advances during this time.

P3 - L27 You report that respondents estimate on average 28/100 neonates treated for suspected sepsis may have “culture-negative sepsis,”

I believe that this needs to be explicitly reported as asked in the survey as “an instinctive guess” and perhaps consider if a median IQR representation of the answers would be of value.

Please consider briefly mentioning alternative explanations for culture-negative cases (e.g. suboptimal sampling volume, non-bacterial etiologies) to avoid over-interpretation that all such cases represent missed infection that would otherwise not be missed by molecular detection.

	The two hypothetical scenarios are a strength and nicely illustrate practice variation. However, you may want to highlight more explicitly that willingness to postpone initiation or to stop antibiotics after a negative rapid test is based on an assumption of results in under 4 hours and 100% sensitivity, which is somewhat unrealistic.<br>P4L23 sentence "a considerable proportion of clinicians still cling to the safety net of 24–48 hours of treatment" could be perceived as a biased comment. Would it make sense to rephrase to something like "a considerable proportion of clinicians prefer to retain the traditional 24–48 hour treatment window as a safety margin" ?<br>The conclusion currently emphasizes(P4L25) "same-shift turnaround, integrated AMR, and robust validation studies;". However, stewardship, culture change, and risk tolerance are also key, since a sizable proportion of respondents would still maintain 24–48 hours of treatment despite rapid negative results.<br>Ethics: you might add explicitly that participation was voluntary and no incentives were provided, if true.

VERSION 1 – AUTHOR RESPONSE

Reviewer: 1
Bernardo Cabral

The survey is well designed, and the findings are of interest. Although space is limited in a research letter, the survey process should be reported in greater detail to improve reproducibility and interpretability. Please describe the sampling frame and recruitment in more detail (which ESPID channels were used, how many individuals were reached and considered eligible, whether reminders were sent, and what measures were taken to prevent duplicate responses), and report the number of complete versus partial responses together with item-level missingness. Given the 7% response rate, include a brief, explicit discussion of nonresponse/selection bias, how the respondent profile (region, setting, specialty) may differ from the target population, and simple diagnostics such as comparing early versus late responders (if feasible). Also, provide clearer information on survey administration (platform, approximate completion time, whether questions were mandatory, any randomization or branching logic, and how a single response per participant was ensured).

We thank the reviewer for their time investment and critical assessment and are pleased to learn they judge the findings of interest. We were allowed limited additional words per this review to elaborate on the relevant points that have been raised and have attempted to action all of them. We have added statements regarding sampling frame, recruitment and frequency of partial responses on page 1. We have performed early and late wave response analysis for all reported outcomes (as reported on page 3 in the revised version) and found no significant differences between waves. Waves were established in 2 ways; (1) was first 70% of respondents versus second 30% of respondents, (2) was a 3 wave division based on when the survey entry was documented relative to the network email to ESPID was sent (resulting in 3 waves) Unfortunately, no data was available regarding survey completion time of individual participants. We did measure these times amongst the pilot team and have added a statement on page 1 with average completion times. Also a statement was added regarding prevention of double entries on page 1, which was not guaranteed, due to the anonymous nature of the survey.

Reviewer: 2
Ioannis Kopsidas

Comments to the Author
Thank you for allowing me to review the research letter “Future perspectives on the use of molecular assays for pathogen identification in neonatal sepsis: a survey study amongst members of the European Society for Paediatric Infectious Diseases” by Groen et al.
The manuscript addresses a gap in neonatal sepsis diagnostics.

We thank the reviewer for their time investment and critical assessment and provide a point-by-point response below.

Study design
P2 L31 April 2024-March 2025 is a very extended period for a survey. Was this sent once in April 2024 and were expecting answers until March 2025? Was it sent more than once? If that is the case, was there a mechanism that ensured that the same person did not answer the survey twice?
It is mentioned that professionals “involved in neonatal sepsis management were invited”. I would assume that ESPID does not have a way of identifying among its members who are involved in management of neonatal sepsis, and the wording of the survey does not deter someone not involved in neonatal sepsis management from answering it. This I believe needs to be under limitations.
Were there any pilot runs of the Survey?

We understand and agree this is confusing and have added several statements to clarify on page 1, stating that the invitation was extended to the entire network (n=2701 at the time) in 3 separate and consecutive emails over the course of the survey period. Due to the anonymous nature of the survey we were not able to remind those who had not started or completed the survey yet. We extended the survey period to generate sufficient participation that would allow us to perform subgroup analyses on several participant characteristics domains, for which we preplanned to include around 200 participants. We explicitized in our invitation that professionals needed to be involved in neonatal sepsis management, but that does not create a full stop for any interested individual who does not necessarily have that expertise, thus participation of people without relevant expertise could not be fully prevented. However, we deem it unlikely that given the details provided in the invitation, such participants were included. Indeed, as the reviewer states, ESPID had no means of identifying the population of interest to our survey.

A statment regarding pilot runs was added on page 1, including a comment on average completion time.

P2 L34 L33 – “quantitative data were analysed descriptively and free-text responses thematically”
a) You do report p values. Briefly describe statistical tests used. Were there any adjustments made?
b) Thematic analysis: How were themes generated, how many coders were involved? Was inter-rater agreement assessed? Provide some information even in one sentence.

We have added a statement (a) on page 1, indicating that all data presented was crude without any adjustments and that Fisher exact tests were used to compare proportions. (b) A statement was also added on the thematic analysis on page 1, indicating that we used reflexive thematic analysis and resolved issues through discussion.

P2 – L35 Consider adding a short statement on how you managed missing data, how many responses were partially vs fully completed and any exclusion criteria

We set a threshold for data missingness of 5% and tested all outcome data to that threshold. Only 1 reported outcome had missingness over that threshold. This involved the question about how much specificity loss participants were willing to accept for using a faster test. 18% did not answer. We added a statement on page 3 regarding the potential cause of this high percentage and that we dealt with it by not actively imputing data. '' Missing responses were not related to any of baseline variables in table 1 and we suspect the complexity of the question led some participants to refrain from answering. As this variable reflects subjective estimates obtained through expert elicitation rather than unobserved true values, missing responses were not imputed.''

P2 L36 – 7% : even though it can be calculated please add number of contacted members.

We agree with te reviewer that this enhances clarity. The total number was 2701. We have added this On page 1.

Limitations : Please discuss in more depth the implications of the low response rate and ESPID-based sampling for selection bias and external validity, especially given the predominance of European, high-resource, academic respondents.

We fully agree with the reviewer that this is very important information and have added a statement in the final paragraph of our letter, stating that the results should be interpreted with cauton due to the limitations as posed by the reviewer. It now reads: ''However, the results should be interpreted cautiously, because of a low response rate, combined with ESPID-based sampling that predominantly reflects European and high resource settings, introducing a substantial risk of selection bias and limiting the external validity.''

Findings overall should be presented with the perspective that can be given, considering the methodology and wording of the survey.

We understand and agree with the reviewers´comment and have modified the letter to reflect that the findings represent only this cohort´s perspective and can not necessarily be generalized to a the wider community, let alone communities outside the one served by the ESPID.

Examples include ´´these findings suggest´´ instead of ''this survey demonstrates'' and ´´participating clinicians´´ instead of ´´clinicians.´´ Also we have added a statement as adressed by the previous comment of this reviewer to stimulate a cautious interpretation of the presented findings, that hopefully puts this into better perspective.

Example: P2L14 "These findings suggest that clinicians recognize the potential of molecular

diagnostics to improve neonatal sepsis management"
This should reflect that these are self-reported expectations from a selected sample rather than evidence that molecular diagnostics actually improve outcomes.

We fully agree with the reviewer and that is why we have modified the wording to reflect a more careful interpretation of the results ´´ These findings suggest that the participating clinicians perceive substantial potential for molecular diagnostics to improve neonatal sepsis NS management´´

Example: P3 L17 It is reported that 43% anticipate full replacement of blood culture within 20 years. However, it should be mentioned that current molecular assays generally do not provide full AMR profiling, genetic markers of resistance are not true phenotypic resistance and and may have limited pathogen range. So, such replacement expects that there will be substantial technological advances during this time.

We fully agree with the reviewer.We have now modified the wording to 'pathogen ID' on page 2 in the hope that this clarifies sufficiently. As can be red in the supplemental blank version of the survey we have submitted previously, the question posed to survey participants was:'' In your opinion, what will be the role of novel diagnostic techniques for pathogen identification for the diagnosis of neonatal sepsis in the coming 20 years of health care practice?''

We hope that by stating ''pathogen identification'' explicitly to survey participants, we have made abundantly clear the aim of the question was not in AMR profiling and that by also stating this in our letter similarly this has become clear. Also, we agree with the reviewer that such replacements will require technological advances, or at the very least adequate assessment of diagnostic performance of assays available today, hence why we set the time threshold in the future.

P3 - L27 You report that respondents estimate on average 28/100 neonates treated for suspected sepsis may have "culture-negative sepsis,"
I believe that this needs to be explicitly reported as asked in the survey as "an instinctive guess" and perhaps consider if a median IQR representation of the answers would be of value.

We have modified the wording from estimated to 'guessed' on page 2 to reflect the original wording of the survey.

Please consider briefly mentioning alternative explanations for culture-negative cases (e.g. suboptimal sampling volume, non-bacterial etiologies) to avoid over-interpretation that all such cases represent missed infection that would otherwise not be missed by molecular detection.
The two hypothetical scenarios are a strength and nicely illustrate practice variation. However, you may want to highlight more explicitly that willingness to postpone initiation or to stop antibiotics after a negative rapid test is based on an assumption of results in under 4 hours and 100% sensitivity, which is somewhat unrealistic.

We fully agree with the reviewer that not all culture negative cases receiving full treatment can automatically be categorized as missed infections. We have added a statement on page 2 ‘’ It should be noted that culture negative cases can reflect either truly missed cases, suboptimal blood collection conditions, non-bacterial etiologies, or the result of unnecessary treatment prolongation.’’

How to classify these children remains truly one of the great conundrums of neonatal sepsis management.

With regards to the case scenarios, to allow an internal conceptualization of how treatment behavior would be modified in case of a faster test result in comparison to using a blood culture pipeline, we aimed to change only the turnaround time condition (molecular assay faster than blood culture) in our case scenarios. If survey participants also had to conceptualize differences in sensitivity and specificity of a molecular assay in comparison to blood culture, this would greatly increase the complexity of the assignment, which is already complex and prone to bias or misinterpretation. The current exercises are not principally different as saying: what if you had a blood culture result in 4 hours. But considering fast turnaround times are the most sought after feature of molecular assays, we implemented this as the main feature in the case scenarios.

Additionally, we would argue that 100% sensitivity of molecular assays in comparison to blood culture is not completely unrealistic (PMID: 40105375) although usually when comparing techniques at least some differences exist (even if performing 2 separate blood culture at the same time from different or the same anatomical locations, such differences exist), but realize the wording in our previous letter version might have made it seem as though this 100% sensitivity was in comparison to ´´clinical/true sepsis´´ instead of in comparison to blood culture. We have modified this to reflect: ‘’ With access to a hypothetical assay offering <4 hour results with identical test performance to BC’’.

Stating that a molecular assay could have 100% sensitivity when using ‘’clinical sepsis’’ as a reference (regardless of the likely debatable definition of clinical sepsis used) would be something different. As the supplemental blank version of the survey shows: ‘’ Assume that test characteristics (sensitivity, specificity) are exactly similar to those of culture based techniques’’ Therefore we hope that the letter now more accurately reflects the text in the survey.

s

P4L23 sentence “a considerable proportion of clinicians still cling to the safety net of 24–48 hours of treatment” could be perceived as a biased comment. Would it make sense to rephrase to something like “a considerable proportion of clinicians prefer to retain the traditional 24–48 hour treatment window as a safety margin”?

We agree with and thank the reviewer this represents better scientific phrasing and have modified accordingly on page 3 following the reviewers’ proposal.

The conclusion currently emphasizes(P4L25) “same-shift turnaround, integrated AMR, and robust validation studies;”. However, stewardship, culture change, and risk tolerance are also key, since a sizable proportion of respondents would still maintain 24–48 hours of treatment despite rapid negative results.

We fully agree and have added these relevant factors on page 3.

Ethics: you might add explicitly that participation was voluntary and no incentives were provided, if true.

VERSION 2 – REVIEW

**REVIEWER NAME**	Cabral, Bernardo
**REVIEWER AFFILIATION**	
**REVIEWER CONFLICT OF INTEREST**	No Conflict of Interest
**AI DISCLOSURE**	No, I have not used AI
**DATE REVIEW RETURNED**	28-Mar-2026

**GENERAL COMMENTS**	The authors have addressed all the comments I made on the previous version.

**REVIEWER NAME**	Kopsidas, Ioannis
**REVIEWER AFFILIATION**	
**REVIEWER CONFLICT OF INTEREST**	No Conflict of Interest
**AI DISCLOSURE**	No, I have not used AI
**DATE REVIEW RETURNED**	30-Mar-2026

**GENERAL COMMENTS**	Thank you for your work in updating the manuscript.<br><br>one small change perhaps here:<br><br>p16L46-47<br>Of 187 respondents accessing the survey, 180 completed the survey completed >70% of<br>items in the outcome domain (response rate 7%).<br><br>Of the 187 respondents who accessed the survey, 180 completed more than 70% of the items in the outcome domain, for an overall response rate of 6.7%.
